# Unconventional microRNA role: Enhancing the human leukocyte antigen class I antigen processing pathway via interacting with a silencer

**DOI:** 10.1002/ctm2.70010

**Published:** 2024-10-22

**Authors:** Yuan Wang, Maria‐Filothei Lazaridou, Theresa Kordaß, Chiara Massa, Christoforos K. Vaxevanis, Stefan Eichmüller, Barbara Seliger

**Affiliations:** ^1^ Institute for Medical Immunology, Martin Luther University of Halle‐Wittenberg Halle Germany; ^2^ Institute of Translational Immunology, Medical School “Theodor Fontane” Brandenburg an der Havel Germany; ^3^ Department of Good Manufacturing Practice (GMP) Development & Advanced Therapy Medicinal Products (ATMP) Design Fraunhofer Institute for Cell Therapy and Immunology (IZI) Leipzig Germany; ^4^ Research Group GMP & T Cell Therapy, German Cancer Research Center (DKFZ) Heidelberg Germany

Dear Editor,

The unconventional functional mechanisms of microRNA (miRNA)‐mediated RNA or protein activation are complex and diverse,^1^ like miRNA binding to AU‐rich elements (ARE)^2^ or competing with RNA‐binding proteins.^3^ Our own data demonstrated that miR‐16 could bind to the coding sequence (CDS) of classical and non‐classical human leukocyte antigen class I (HLA‐I) molecules, thereby inducing their expression.^4^ However, the interaction of silencers with miRNAs has not yet been investigated. Silencer features include a high GC content,^5^ DNase hypersensitivity sites^6^ and H3K27me3 regions.^7^ Here we identified for the first time that miR‐155‐5p can directly bind a silencer in the 3′untranslated region (3′UTR) of TAP‐binding protein (tpn) thereby increasing the HLA‐I surface expression.

Using miRNA trapping by RNA in vitro affinity purification (miTRAP),^8^ in silico analyses and molecular experiments, we identified miR‐155‐5p targeting of tpn 3′ UTR in melanoma cells affecting tpn and cell surface HLA‐I expression, which has also clinical relevance. Interestingly, upon deletion of the predicted binding site within tpn 3´UTR (Figure 1A), luciferase (luc) reporter assays indicated higher relative luc activity of the wild type (wt) compared to the del 3′UTR in HEK293T cells (Figure 1B), which is opposite to the conventional function of miRNAs leading to a downregulation. Overexpression of miR‐155‐5p in three melanoma cell lines (Figure 1C) increased their tpn messenger RNA (mRNA) (Figure 1D) and protein levels (Figure 1E,F). This upregulation was specific for tpn since the mRNA of programmed death ligand 1 (PD‐L1), another target of miR‐155‐5p,^9^ was downregulated in the miR‐155‐5p‐transfected MZ‐Mel2 cell line (Figure 1G). Despite the overall expression levels of the HLA‐I heavy chain (HC) were not altered in the miR‐155‐5p transfectants (Figure 1F,H), a tpn‐mediated upregulation of the HLA‐ABC and HLA‐BC surface antigens was found on FM81 and MZ‐Mel2 cells (Figure 1I,J), but not on FM3 cells, which might be probably due to the high HLA‐I surface expression when compared to FM81 and MZ‐Mel2 cells (Figure 1F). Actinomycin D treatment revealed a significant increase in the tpn mRNA half‐life in FM81 miR‐155‐5p transfectants (Figure 1K). Using a CD107a degranulation assay, a reduced NK cell‐mediated cytotoxicity against miR‐155‐5p transfected MZ‐Mel2 cells expressing increased HLA‐I surface antigens was shown by lower numbers of CD107a‐positive NK cells (Figure 1L).

**FIGURE 1:**
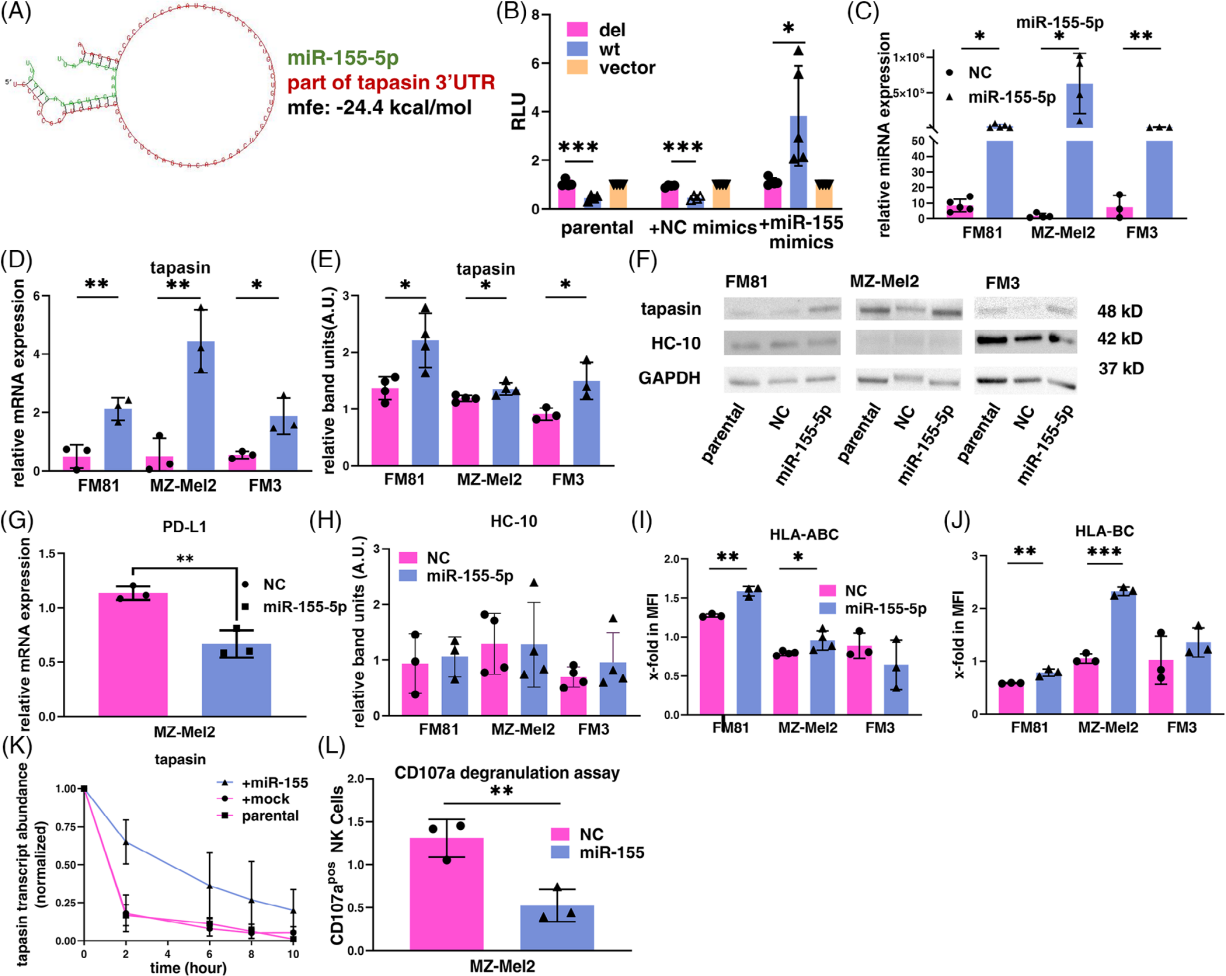
MiR‐155‐5p‐mediated upregulation of tapasin enhances the human leukocyte antigen class I (HLA‐I) cell surface expression. (A) The binding site predicted tpn 3′untranslated region (3′UTR) (red) and miR‐155‐5p (green) interactions, including sequence alignment, secondary structure and free energy (mfe = −24.4 kcal/mol) were obtained using the RNAhybrid online database. (B) MiR‐155‐5p and tpn direct interaction identified by dual luciferase reporter assay using HEK293T cells and pmiR‐GLO plasmid. The Firefly luciferase (FFL) activities were normalized to Renilla luciferase activities to give the relative light units (RLU). The data represent the mean ± SD of three biological replicates upon their normalization to parental cells. (C, D) RT‐qPCR was performed to determine the mRNA expression of miR‐155‐5p in three metastatic melanoma cell lines after transfection of miR‐155‐5p mimic or miR mimic NC for 48 hrs. The data represent the mean ± SD of three independent biological replicates upon their normalization to parental cells. (E, F) To determine the expression of the tpn protein after transfection with miR‐155‐5p or NC, Western blot analyses were performed. The relative band intensities (A.U., arbitrary units) of each group were compared to that of the corresponding parental melanoma cells and normalized to the corresponding GAPDH signals (mean ± SD, *n* = 3 biological replicates). (G) RT‐qPCR was performed to determine PD‐L1 mRNA expression after transfection of the metastatic melanoma cell line MZ‐Mel2 with miR‐155‐5p mimic or NC mimic for 48 hrs. Data represent the mean ± SD of three independent biological replicates upon their normalization to parental cells. (H) Western blot analyses were performed to explore the expression of the HLA‐I heavy chain after transfection with miR‐155‐5p or NC. The relative band intensities (A.U., arbitrary units) of each group were compared with the corresponding parental melanoma cells and normalized to the corresponding GAPDH signals (mean ± SD, *n* = 4 biological replicates). (I, J) Flow cytometry was used to determine the HLA‐I surface expression. For staining melanoma cells, Abs directed against HLA‐ABC and HLA‐BC were employed. The data were presented as mean fluorescence intensities (MFI) to parental cells (mean ± SD, *n* = 3 biological replicates). (K) The Act D mRNA stability assay determines the half‐life of the tpn mRNA expression at different treatment time points after transfection as previously described using RT‐qPCR normalized to the mRNA expression of ALAS1 (mean ± SD, *n* = 3 biological replicates). (L) The CD107a degranulation assay was employed to determine the miR‐155‐5p‐mediated effect on HLA‐I cell surface expression in association with NK cell cytotoxicity (mean ± SD, *n* = 3 biological replicates). *p < .05, **p < .01 and ***p < .001.

The overall survival (OS) analysis of 214 metastatic melanoma cases with patients’ outcomes demonstrated a positive correlation of miR‐155 (miR‐155HG) (Figure 2A), tpn (Figure 2B) and HLA‐A (Figure 2C) expression levels with the OS of patients, thereby confirming ours in vitro experiments. Similar results were obtained by bioinformatics analyses of all 444 cases or 63 cases of distant metastatic melanoma from the “TCGA Skin Cutaneous Melanoma (SKCM)” dataset (Figure 2D,E). Furthermore, a strong positive correlation between miR‐155 expression and the frequency of CD8^+^ T cells was found in this dataset using the CIBERSORT web tool (Figure 2F,G).

**FIGURE 2:**
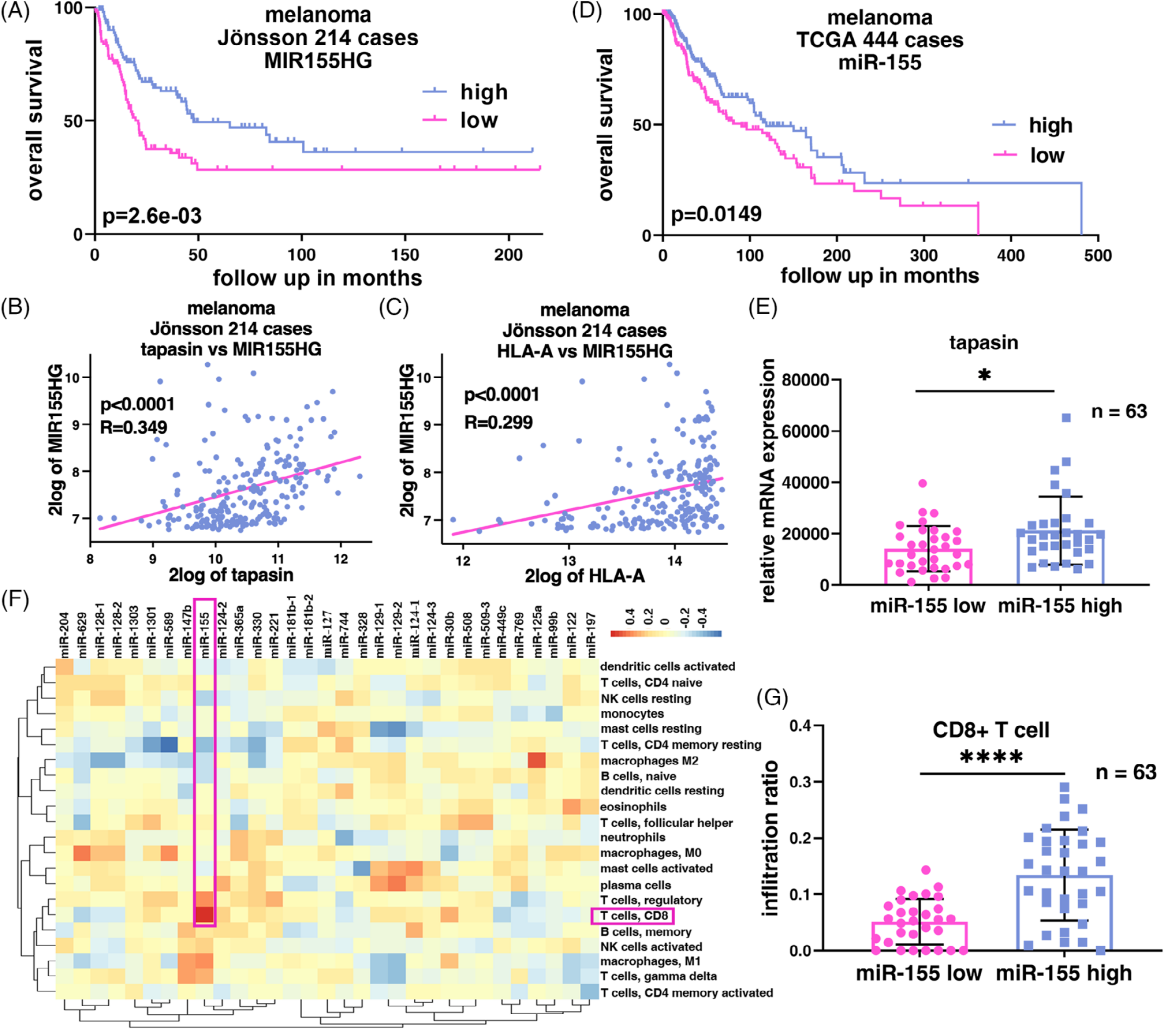
Clinical significance of miR‐155‐5p and its relationship with immune cell infiltration. (A–C) The dataset (GSE65904) from the "R2: Genomics Analysis and Visualization Platform (http://r2.amc.nl)” web tool was used to evaluate the patient's OS determined by the Kaplan Meier estimation curve and correlations of MIR155HG expression with tpn or HLA‐A expression, respectively. (D) The TCGA‐SKCM dataset was used to correlate the patients’ OS with miR‐155 expression. (E) 63 metastatic melanoma cases with available RNA and miRNA seq data in the TCGA‐SKCM dataset were employed to verify the association between tpn and miR‐155. (F, G) Using the dataset from E, the role of miRNA expression on immune cell composition and the relationship between CD8^+^ T cell infiltration and the expression of miR‐155 were analyzed by the generation of a heat map. (mean ± SD, *n* = 3 biological replicates). *p < .05, **p < .01 and ****p < .0001.

Subsequently, in silico analysis of the miRNA binding site sequence in the 3′ UTR of tpn revealed a high GC content and a DNase hypersensitivity site (DHS) suggesting its role as a part of a silencer (Table S1 and Figure S1). Three sequences (sil1, sil3, sil4) upstream of the binding site, an AU‐rich element predicted via the ARE site web tool and the potential GA‐, AU‐ and GC‐rich areas within the miR‐155‐5p binding site were deleted in the tpn 3´UTR (Figure 3A and Figure S2A) and cloned into the miR‐GLO‐vector since the miRNA‐mediated activation^10^ is linked to the binding to specific sequence elements. The luc reporter assay revealed that in comparison to the wt 3′UTR, the transfection of AREdel, AUdel, GCdel, or GAdel vectors into the HEK293T cells reverted the suppressive effects in the negative control (NC), parental cells and the positive effect in miR‐155‐5p transfectants. Since these elements belong to the miR‐155‐5p binding site, their deletions have disrupted the binding site leading to the loss of the positive miR‐155‐5p function on tpn after transfection confirming these elements represent parts of the miR‐155‐5p binding site and the core part of a silencer. Whereas in both NC and parental cells, the repressive effects of sil1del, sil3del or sil4del groups were similar to the wt group, all sil groups in the miR‐155‐5p transfected cells have positive, but much lower effects than in the wt group (Figure 3B) suggesting the disruption of secondary mRNA structures by the partial sequence deletions (Figure S2B–K) are involved in this enhancement.

**FIGURE 3:**
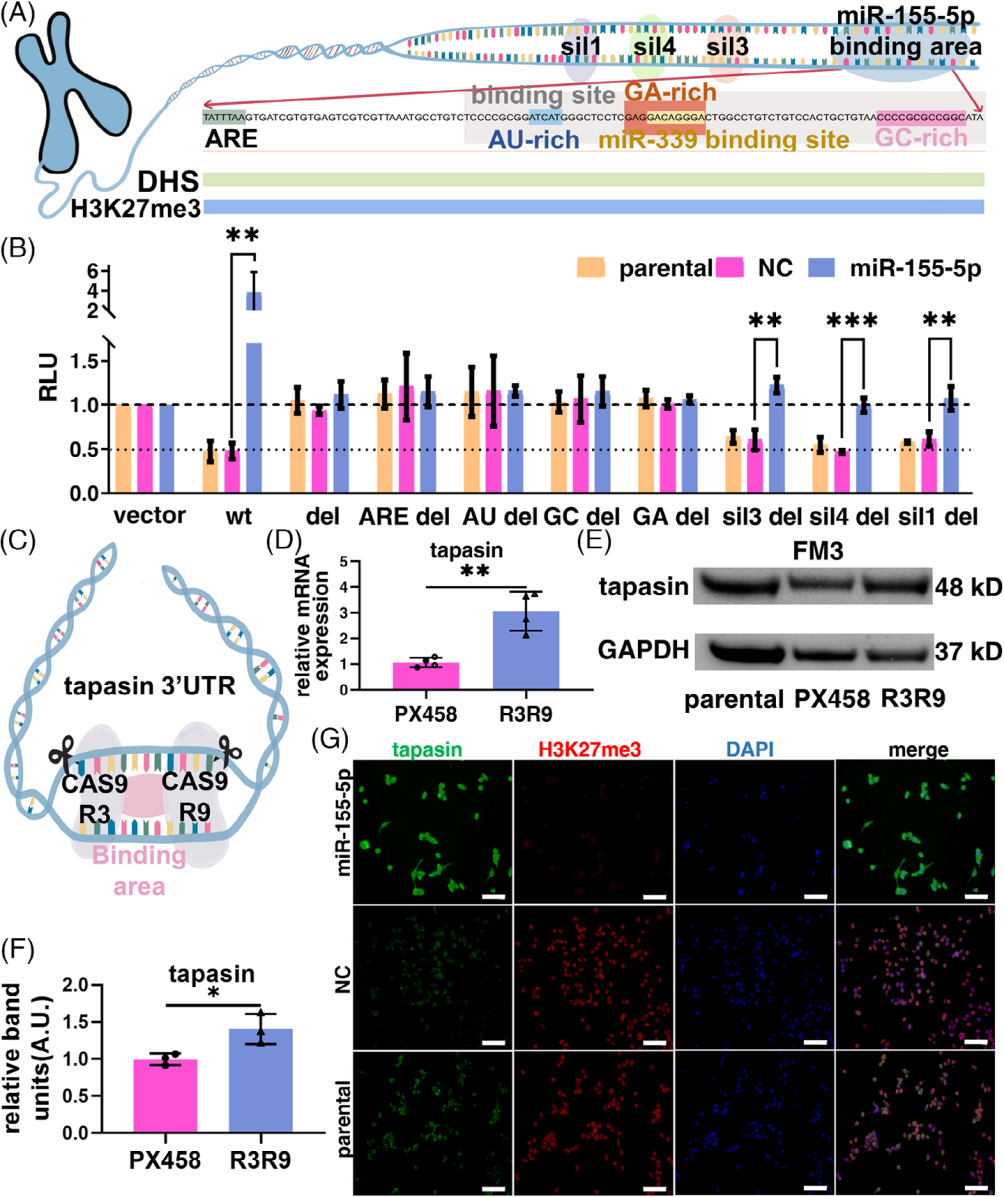
Characterization of the miR‐155‐5p binding site on the 3′untranslated region (3′UTR) of tapasin as a silencer. (A) Simulation diagram for the sequence of the miR‐155‐5p binding site, elements around this binding site and characteristics of this sequence. (B) Deletion of the binding sites and of some possible functional elements to investigate the activity of this area using luciferase assays using pmiR‐GLO vector after transfection with miR‐155‐5p mimics or negative control mimics (mean ± SD, *n* = 3 biological replicates). (C) Simulation diagram of the knockout sequence of the binding site area using CRISPR/Cas9 system. (D) RT‐PCR was performed to investigate the tpn mRNA expression after knocking out the binding site via vector R3 and R9 (R3R9) as well as using the transfection with the PX458 empty vector “the pSpCas9 (BB)−2A‐GFP plasmid” (PX458) as negative control (mean ± SD, *n* = 3 biological replicates), respectively. (E, F) The Western blot analyses were performed to determine the expression of the tpn protein after knocking out the binding site via CRISPR/Cas9. The relative band intensities (A.U., arbitrary units) of each group were compared to that of the corresponding parental FM3 melanoma cells and normalized to the corresponding GAPDH signals (mean ± SD, n = 3 biological replicates). (G) Immune cytofluorescence was used to investigate the correlation between tpn (green) and H3K27me3 (red) after transfection with miR‐155‐5p mimics and NC mimics. The scale is 125 µm. *p < .05, **p < .01 and ***p < .001.

In addition, a CRISPR/Cas9‐mediated deletion of the miR‐155‐5p binding site was generated in the FM3 melanoma cell line upon transfection of modified PX458 (Figure 3C and Figure S2A). Sanger sequencing (data not shown) and PCR amplification (Figure S2L) demonstrated the successful deletion of the miR‐155‐5p binding site (R3R9) resulting in an upregulation of tpn mRNA and protein levels when compared to the mock vector (PX458) (Figure 3D–F). As shown in Figure 3G, the tpn expression (green) was higher in the miR‐155 group than in the other two groups, while the expression of H3K27me3 (red) was inversely correlated due to direct or indirect binding of tpn to H3K27me3 as shown by immunoprecipitation (Figure S2M), which strengthen the evidence that the binding site is part of a silencer.

Finally, to exclude that the miRNA‐mediated activation of tpn was caused by RBPs, the four proteins HNRNPL, HNRNPC, IGF2BP1 and IGF2BP3, known to bind to the tpn 3′UTR, were explored. Compared to HNRNPC and HNRNPL, the IGF2BP1 and IGF2BP3 binding sites overlap with that of miR‐155‐5p (Figure S3A–D). Interestingly, the miR‐155‐5p binding site is a part of the IGF2BP1 and IGF2BP3 potential binding sites containing a GC‐ and an AU‐rich element (Figure S3E). Concentration gradient silencing assays revealed an interaction of miR‐155‐5p and these RBPs (Figure S3F–H).

This study proposes a new unconventional function of miRNAs that enhances target transcription through binding a silencer thereby activating the downstream pathway(s) (Figure 4). Using miR‐155‐5p as a model, this is the first report (i) identifying a silencer in the tpn 3′UTR, which (ii) directly interacts with a non‐coding RNA and (iii) has clinical relevance. These data extend miRNAs’ functions and add new insights to our knowledge of silencers.

**FIGURE 4:**
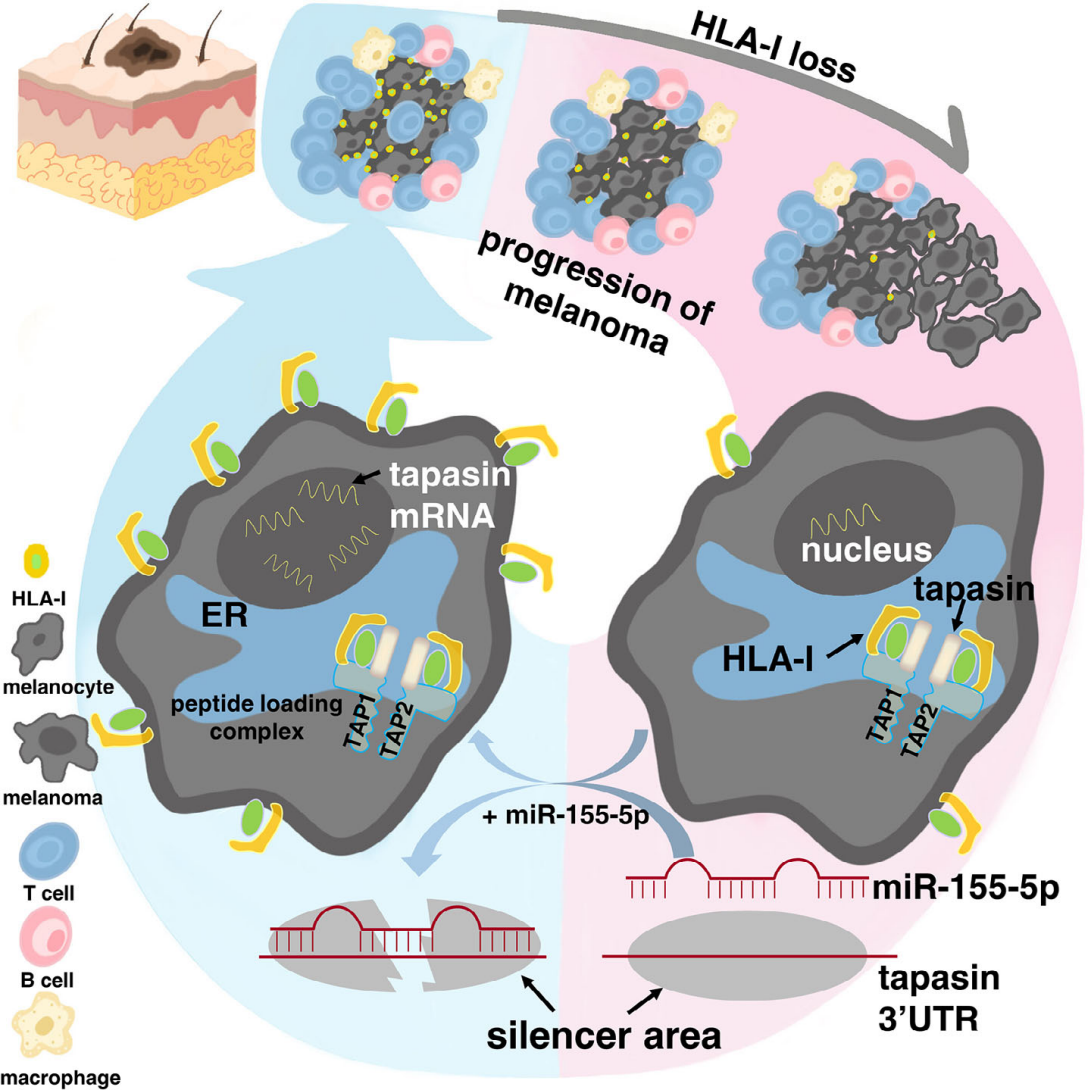
MiR‐155‐5p‐mediated inhibition of the silencer area and activation of the antigen processing and presentation pathway. During the progression of melanoma, human leukocyte antigen class I (HLA‐I) surface expression is gradually lost accompanied by a decreased expression of tpn. Upon transfection into melanoma cell lines, miR‐155‐5p binding to a silencer sequence in the tpn 3′untranslated region (3′UTR) disrupts the function of the silencer, thereby promoting the transcription. The upregulation of tpn enhances the antigen processing and presentation pathway, thereby leading to increased HLA‐I surface antigens for immune cell recognition.

## AUTHOR CONTRIBUTIONS

Conceptualization: Yuan Wang, Barbara Seliger, Theresa Kordaß and Stefan Eichmüller. Methodology: Yuan Wang, Maria‐Filothei Lazaridou, Chiara Massa, Theresa Kordaß, Christoforos K. Vaxevanis. Investigation: Yuan Wang, Maria‐Filothei Lazaridou and Chiara Massa. Visualization: Yuan Wang, Maria‐Filothei Lazaridou and Chiara Massa. Funding acquisition: Barbara Seliger and Stefan Eichmüller; Writing—original draft: Yuan Wang and Barbara Seliger; Writing—review & editing: Yuan Wang, Barbara Seliger, Theresa Kordaß, Stefan Eichmüller, Maria‐Filothei Lazaridou, Chiara Massa, Christoforos K. Vaxevanis.

## CONFLICT OF INTEREST STATEMENT

The authors declare no conflict of interest.

## FUNDING INFORMATION

The work was funded by grants of the Deutsche Krebshilfe, grant 341025929 (Barbara Seliger) and the Deutsche Forschungsgemeinschaft, grant number: SE581/33‐1.

## Supporting information

(A) The sequence of the tpn 3′UTR was examined by ENCODE in the *Homo sapiens suprapubic skin tissue* dataset. The binding site area of miR‐155‐5p is a GC‐rich region and has DNase hypersensitivity sites as well as peaks of H3K27me3. (B) 3095 bp silencer sequence was identified by using the silencer database SilencerDB. In addition, the miR‐155‐5p binding site located in the 3′UTR of tpn (ENST00000434618.2) is a part of this silencer.

(A) The deletion regions of sil1, sil3 and sil4 are marked by red, green, and pink boxes, respectively. The yellow box highlights the miR‐155‐5p binding site on the 3′UTR. The two sequences in red are the target sequences for clipping sites of CRISPR/Cas9, respectively. (B–K) The secondary structure of tpn 3′UTR was predicted using the UNAFold Web server (http://www.unafold.org/). RNA folding results show structure 1 folding bases 1–2065 of NM_001410875 1 Homo sapiens TAP binding protein [T Initial ΔG = −697.50]. (B–C) The secondary structures overlap with sil1 deletion area (red). (D–G) The secondary structures overlap with sil3 deletion area (red). (H–K) The secondary structures overlap with sil4 deletion area (red). (L) In the R3R9 group, approximately 128 bp sequences were knocked out via CRISPR/Cas9 compared to PX458 and parental groups (814 bp). (M) The immunoprecipitation and Western blot analyses were performed as described in Materials and Methods to determine the expression of the tpn protein after immunoprecipitation and transfection with miR‐155‐5p or NC in FM81 melanoma cell line. The input group, not subjected to immunoprecipitation served as a control. Ip was the immunoprecipitation group and the supernatant represented the residual liquid after separation and precipitation.

(A–D) The binding sites of the four proteins on tpn 3′UTR were predicted using RBPsuite. The red dots represent the binding sites with higher probability. The position circled in red is the binding site for miR‐155‐5p. (A, B) the predicted results for HNRNPC and HNRNPL. (C, D) Some of the predicted binding sites of IGF2BP1 and IGF2BP3 overlap with the miR‐155‐5p binding site. (E) The predicted binding site 1 (blue) and site 2 (pink) of IGF2BP1 and IGF2BP3 overlap with the miR‐155‐5p binding site (red box) on the tpn 3′UTR. The black boxes are GC‐ and AU‐rich areas. (F–H) The effects of concentration gradient silencing of IGF2BP1 and IGF2BP3 on the binding site sequence were investigated using luciferase assays after transfection of siRNAs (IGF2BP1, IGF2BP3, negative control) and cloned miR‐GLO vector into HEK 293T cell lines (mean ± SD, *n* = 3 biological replicates). *p < .05 and **p < .01.

Supporting Information

## Data Availability

The miRNA seq data are available in the GEO database (accession numbers GSE240665). The mass spectrometry proteomics data have been deposited to the ProteomeXchange Consortium via the PRIDE partner repository with the dataset identifier PXD045056. Other data underlying this article are available in ZENODO (https://doi.org/10.5281/zenodo.10007575 and https://doi.org/10.5281/zenodo.8315517) or available in the manuscript or the supplementary materials.
