# Supplementary figures and images for: Unconventional microRNA role: Enhancing the human leukocyte antigen class I antigen processing pathway via interacting with a silencer
Source: Clin Transl Med. 2024 Oct 22;14(10):e70010. doi: 10.1002/ctm2.70010 (PMC11496566; doi:10.1002/ctm2.70010)

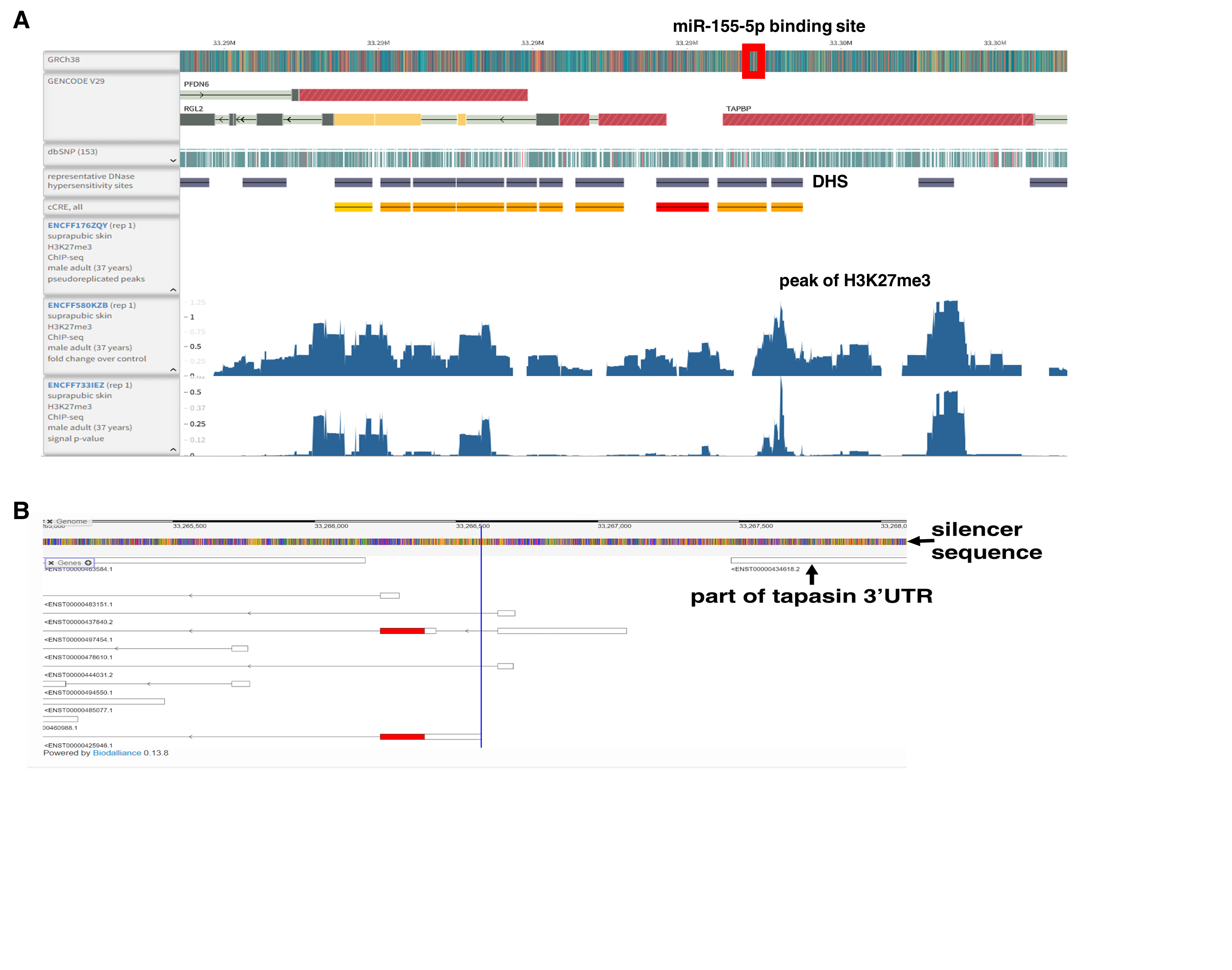

Supplement: Supplementary file 1 — (A) The sequence of the tpn 3′UTR was examined by ENCODE in the Homo sapiens suprapubic skin tissue dataset. The binding site area of miR‐155‐5p is a GC‐rich region and has DNase hypersensitivity sites as well as peaks of H3K27me3. (B) 3095 bp silencer sequence was identified by using the silencer database SilencerDB. In addition, the miR‐155‐5p binding site located in the 3′UTR of tpn (ENST00000434618.2) is a part of this silencer. [file CTM2-14-e70010-s003.tif]

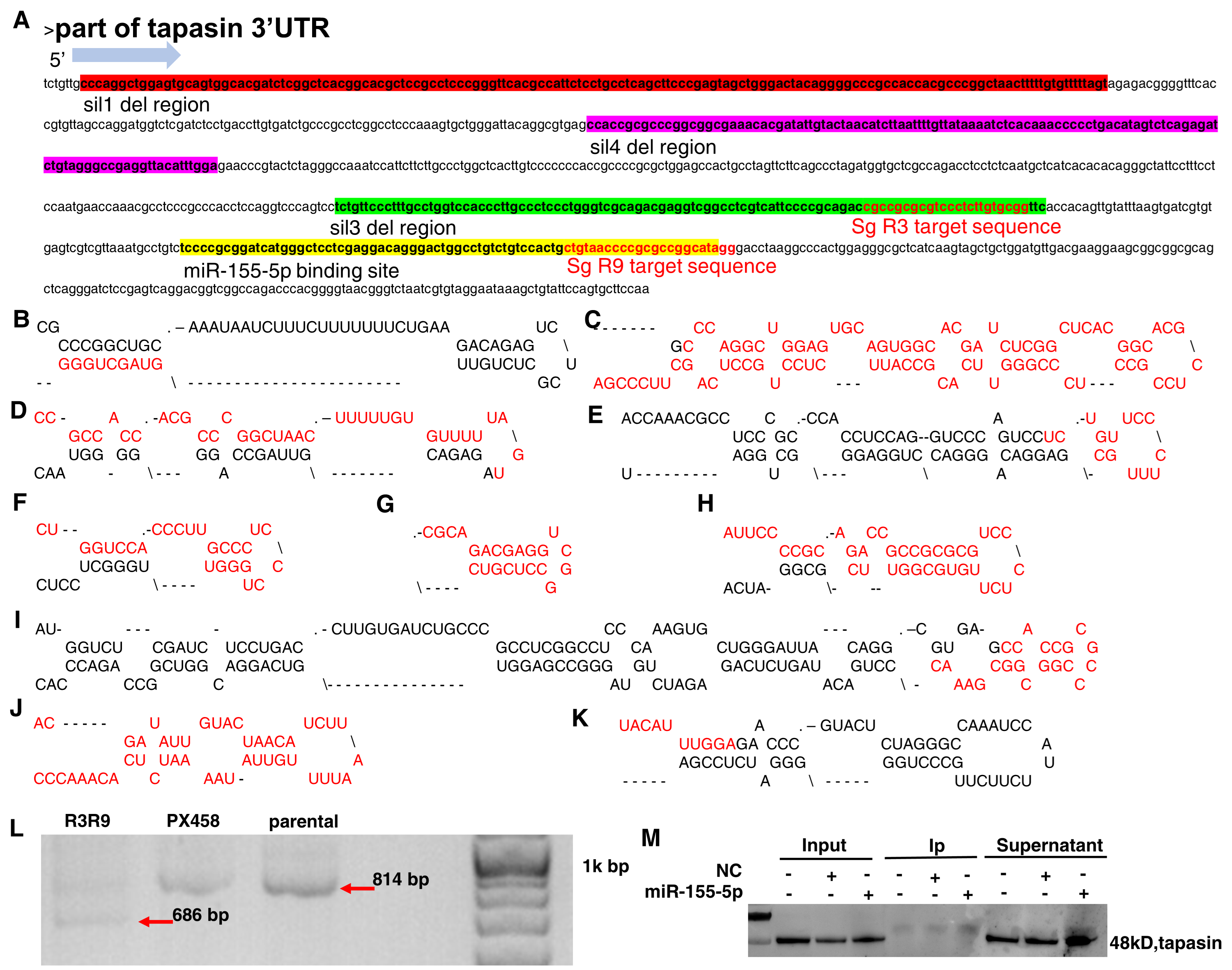

Supplement: Supplementary file 2 — (A) The deletion regions of sil1, sil3 and sil4 are marked by red, green, and pink boxes, respectively. The yellow box highlights the miR‐155‐5p binding site on the 3′UTR. The two sequences in red are the target sequences for clipping sites of CRISPR/Cas9, respectively. (B–K) The secondary structure of tpn 3′UTR was predicted using the UNAFold Web server (http://www.unafold.org/). RNA folding results show structure 1 folding bases 1–2065 of NM_001410875 1 Homo sapiens TAP binding protein [T Initial ΔG = −697.50]. (B–C) The secondary structures overlap with sil1 deletion area (red). (D–G) The secondary structures overlap with sil3 deletion area (red). (H–K) The secondary structures overlap with sil4 deletion area (red). (L) In the R3R9 group, approximately 128 bp sequences were knocked out via CRISPR/Cas9 compared to PX458 and parental groups (814 bp). (M) The immunoprecipitation and Western blot analyses were performed as described in Materials and Methods to determine the expression of the tpn protein after immunoprecipitation and transfection with miR‐155‐5p or NC in FM81 melanoma cell line. The input group, not subjected to immunoprecipitation served as a control. Ip was the immunoprecipitation group and the supernatant represented the residual liquid after separation and precipitation. [file CTM2-14-e70010-s004.tif]

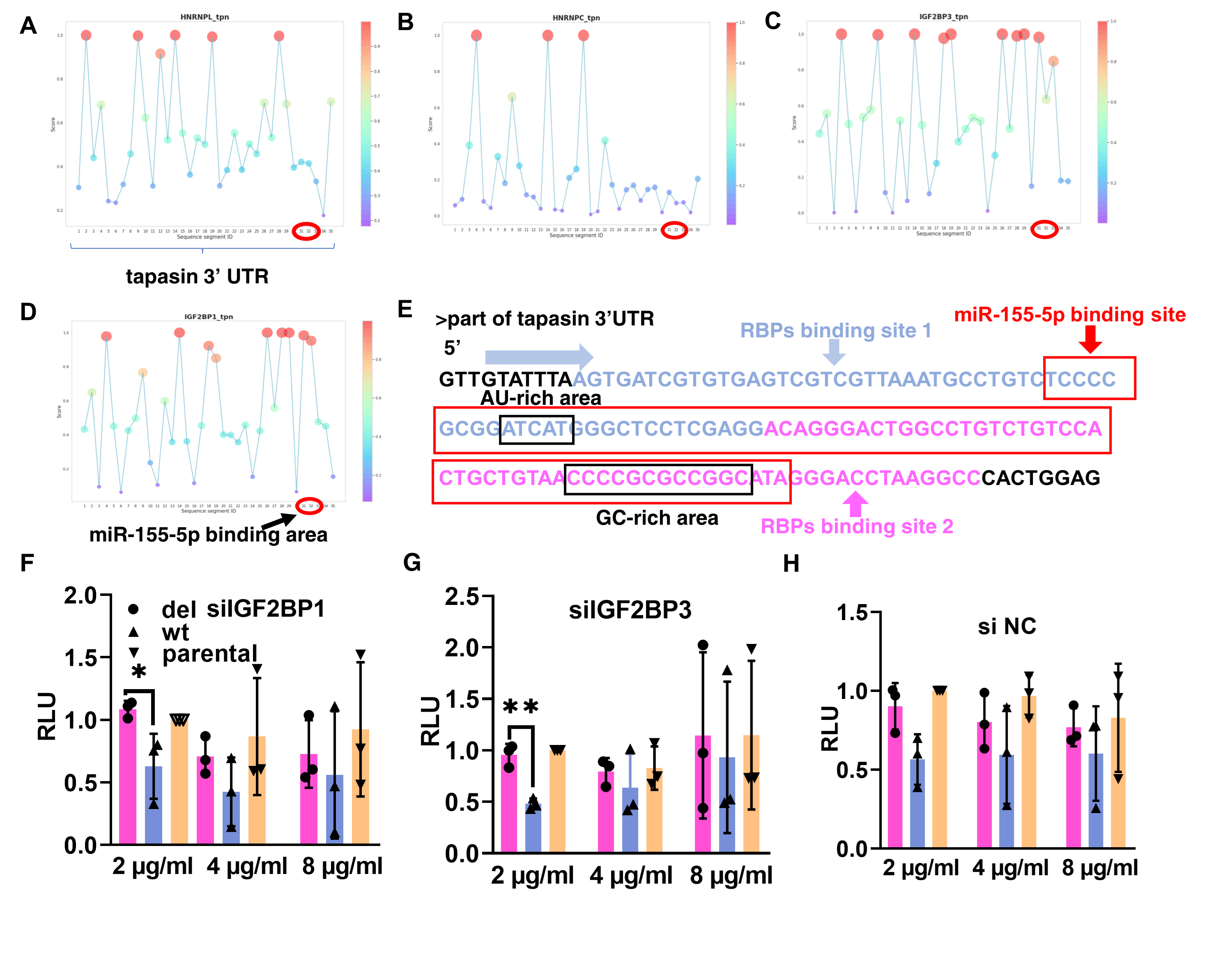

Supplement: Supplementary file 3 — (A–D) The binding sites of the four proteins on tpn 3′UTR were predicted using RBPsuite. The red dots represent the binding sites with higher probability. The position circled in red is the binding site for miR‐155‐5p. (A, B) the predicted results for HNRNPC and HNRNPL. (C, D) Some of the predicted binding sites of IGF2BP1 and IGF2BP3 overlap with the miR‐155‐5p binding site. (E) The predicted binding site 1 (blue) and site 2 (pink) of IGF2BP1 and IGF2BP3 overlap with the miR‐155‐5p binding site (red box) on the tpn 3′UTR. The black boxes are GC‐ and AU‐rich areas. (F–H) The effects of concentration gradient silencing of IGF2BP1 and IGF2BP3 on the binding site sequence were investigated using luciferase assays after transfection of siRNAs (IGF2BP1, IGF2BP3, negative control) and cloned miR‐GLO vector into HEK 293T cell lines (mean ± SD, n = 3 biological replicates). *p < .05 and **p < .01. [file CTM2-14-e70010-s002.tif]
