# Supplementary material for: Unconventional microRNA role: Enhancing the human leukocyte antigen class I antigen processing pathway via interacting with a silencer
Source: Clin Transl Med. 2024 Oct 22;14(10):e70010. doi: 10.1002/ctm2.70010 (PMC11496566; doi:10.1002/ctm2.70010)
Supplement: Supplementary file 4 — Supporting Information [file CTM2-14-e70010-s001.docx]

**Supporting information:**

| **chromosome** | **start** | **end** | **cell line** | **tissue** | **organ** | **species** | **method** | **nearest gene** | **regulatory gene** |
| --- | --- | --- | --- | --- | --- | --- | --- | --- | --- |
| chr6 | 33265041 | 33268136 | fetal intestine small | small intestine | intestine | home sapiens | SVM | PFDN6 | TAPBP |
| chr6 | 33265041 | 33268136 | h1 derived neuronal progenitor cultured cells | brain | brain | home sapiens | SVM | PFDN6 | TAPBP |
| chr6 | 33265041 | 33268136 | gastric | stomach | stomach | home sapiens | SVM | PFDN6 | TAPBP |

**Table S1. Characteristics of the silencer sequence including the miR-155-5p binding site in three different cell lines of distinct tissue origin**
